# Successful Treatment of Chidamide and Cyclosporine for Refractory/Relapsed Angioimmunoblastic T Cell Lymphoma With Evans Syndrome: A Case Report With Long-Term Follow-Up

**DOI:** 10.3389/fonc.2020.01725

**Published:** 2020-08-27

**Authors:** Fang Zhu, Qiuhui Li, Huaxiong Pan, Yin Xiao, Tao Liu, Xinxiu Liu, Juan Li, Gang Wu, Liling Zhang

**Affiliations:** ^1^Cancer Center, Union Hospital, Tongji Medical College, Huazhong University of Science and Technology, Wuhan, China; ^2^Department of Pathology, Union Hospital, Tongji Medical College, Huazhong University of Science and Technology, Wuhan, China

**Keywords:** angioimmunoblastic T cell lymphoma, chemotherapy, Evans syndrome, chidamide, cyclosporine, case report

## Abstract

**Background:**

Refractory/relapsed angioimmunoblastic T cell lymphoma (AITL) with Evans syndrome is a very rare condition with a poor prognosis. There is no evidence-based treatment strategy for refractory/relapsed AITL with Evans syndrome.

**Case Presentation:**

A 51-year-old female was admitted to our hospital with multiple enlarged bilateral cervical lymph nodes, more than 1 week-long chest distress, and night sweats in July 2014. An excision biopsy of the left cervical enlarged lymph node revealed AITL. However, the patient showed resistance to the first-line chemotherapy for AITL and was diagnosed with refractory AITL. Complete remission was achieved after the salvage treatment with the combination of chemotherapy, radiotherapy, and immunomodulatory agent lenalidomide. Unfortunately, 12 months later, the patient suffered from disease progression and was diagnosed as refractory/relapsed AITL with Evans syndrome according to the laboratory findings and imaging. With the diagnosis of refractory/relapsed AITL with Evans syndrome, the patient received the first-line treatment for Evans syndrome including prednisone and intravenous immunoglobulin. The response to the first-line treatment for Evans syndrome was poor. The combination regimen of chidamide (30 mg, po, biw) and cyclosporine were administrated considering the treatment targeting simultaneously both refractory/relapsed AITL and Evans syndrome. The efficacy evaluation was complete remission. The last follow-up of the patient was April 30th, 2020, and no evidence of disease progression was observed. The overall survival of the patient was more than 70 months.

**Conclusion:**

The treatment for refractory/relapsed AITL combined with Evans syndrome remains challenging to patients and physicians. The combination of chidamide and cyclosporine may be an effective and tolerable regimen for the intractable AITL with Evans syndrome case and more observations are necessary to identify the efficacy and safety in the future.

## Introduction

Peripheral T-cell lymphoma (PTCL) is a group of heterogeneous diseases including more than twenty subtypes, with low incidence and unique pathobiology (1). Angioimmunoblastic T-cell lymphoma (AITL) is the second most common subtype of PTCL with an aggressive clinical course and poor prognosis, which accounts for about 15–20% of PTCL cases, 1–2% of all non-Hodgkin lymphomas (NHL) (2). Many AITL patients present in advanced stage at the initial diagnosis. The presentations of immunologic hyperactivation are frequently observed in AITL patients including polyclonal hypergammaglobulinemia, hemolytic anemia, and the presence of various autoantibodies (3).

The standard therapy for AITL patients remains unclear. According to the newest National Comprehensive Cancer Network (NCCN) practice guideline of T-cell lymphoma, participating in clinical trials or receiving chemotherapy with CHOP (cyclophosphamide, adriamycin, vincristine, and prednisolone) or CHOP-based regimens with or without radiotherapy are the options of the first-line therapy for AITL patients (4). However, AITL showed a poorer response to conventional chemotherapy than that of B-cell lymphoma. Only less than 50% of patients achieved a complete response, and disease relapse occurred commonly in the majority of AITL patients after a short-term remission. Unfortunately, the refractory/relapsed AITL patients usually showed poor response to the salvage chemotherapy (5). Therefore, the treatment of relapsed/refractory AITL remains a challenge for physicians.

Evans syndrome is a rare autoimmune disease that was described by Robert Evans in 1951 (6). It is characterized by the presence of simultaneous or sequential autoimmune hemolytic anemia and immune thrombocytopenia with a positive direct anti-human globulin test. Evans syndrome can be classified into primary or secondary based on the presence of basic diseases such as hematological malignancy, rheumatic disorder, and so on. Secondary Evans syndrome showed a poorer prognosis with the median survival of 1.7 years than that of primary Evans syndrome (7). There is no evidence-based therapy for secondary Evans syndrome accompanied by lymphoma because of the rarity.

To our knowledge, this is the first report of refractory/relapsed AITL with Evans syndrome successfully treated with the combination of chidamide and cyclosporine without severe adverse effect.

## Case Presentation

**FIGURE 1:**
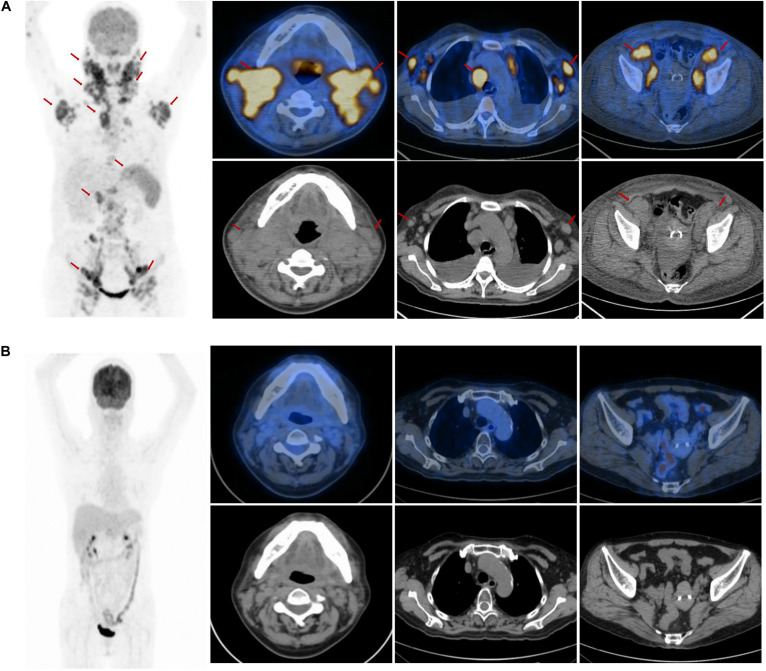
The positron emission tomography-computed tomography (PET-CT) scan at the first admission and the end of the salvage treatment. **(A)** The PET-CT scan at the first admission revealed elevated FDG uptake in multiple enlarged lymph nodes in the bilateral parotid gland, bilateral cervical, bilateral axillary, mediastinal, retroperitoneal, bilateral pelvic, bilateral inguinal regions, and in the nasopharynx, tonsil and spleen (arrow). **(B)** The PET-CT scan showed no elevated FDG uptake lesions at the end of the salvage treatment.

A 51-year-old female was admitted to our hospital with multiple enlarged cervical lymph nodes, more than 1 week-long chest distress, and night sweats in July 2014. The patient had no personal or family medical history of a malignant neoplasm. A computed tomography (CT) scan revealed multiple enlarged cervical lymph nodes. An excision biopsy of the left cervical enlarged lymph node revealed AITL. The immunohistochemistry showed that the tumor cells were positive staining for CD3, CD10, BCL6, CXCL13, PD1, CD21, and negative staining for CD20, CD30, LMP1, MUM1, Cyclin D1, as well as high proliferation (30% of Ki-67 staining positive cells). The Epstein-Barr virus-encoded RNA was positive. A further ^18^FDG positron emission tomography-computed tomography (PET-CT) scan confirmed that multiple FDG-avid enlarged lymph nodes in the bilateral cervical, bilateral axillary, mediastinal, retroperitoneal, bilateral pelvic, and bilateral inguinal regions (Figure 1A). PET-CT scan also showed pathological uptake in the bilateral parotid gland, nasopharynx, tonsil, and spleen, which were considered as lymphoma infiltration. Bone marrow biopsy was negative for lymphoma. The patient was diagnosed with stage IVB AITL.

Two cycles of CHOP (cyclophosphamide 750 mg/m^2^, day 1; adriamycin 50 mg/m^2^, day 1; vincristine 2 mg, day 1; prednisone 100 mg, days 1–5) were administrated considering the poor performance status of the patient at the time of the initial diagnosis. After 1 cycle of chemotherapy with CHOP, the cervical enlarged lymph nodes reduced. It was unexpected that the cervical lymph nodes increased rapidly 12 days after the second cycle of chemotherapy. We changed the third cycle chemotherapy regimen to a biweekly regimen GEMOX (gemcitabine 1000 mg/m^2^, days 1 and 15; oxaliplatin 100 mg/m^2^, days 1 and 15) considering the rapid proliferation of tumor. However, the cervical enlarged lymph nodes reduced after chemotherapy and increased again after 11 days of chemotherapy. Herein, the patient was diagnosed as the refractory AITL because of the poor response to the first-line treatment. Therefore, we continued two cycles of chemotherapy with CHOEP (cyclophosphamide 750 mg/m^2^, day 1; adriamycin 50 mg/m^2^, day 1; vincristine 2 mg, day 1; etoposide 750 mg/m^2^, days 1–3; prednisone 100 mg, days 1–5). The CT scan revealed that the lymph nodes reduced significantly in mediastinal, retroperitoneal, bilateral pelvic, and bilateral inguinal regions, but the cervical lymph nodes increased slightly. Considering that AITL was highly aggressive and refractory, the addition of lenalidomide (20 mg, po, days 1–14, q21d) to CHOEP for 3 cycles and simultaneous intensity-modulated radiation therapy with the dose of 50 Gy in 25 fractions were given with adequate support treatment of granulocyte colony-stimulating factor, thrombopoietin. The clinical target volume of radiotherapy included the regions of the bilateral parotid gland, bilateral cervical lymph nodes. The response was complete remission at the end of the salvage treatment, which was confirmed by the PET-CT scan (Figure 1B). She refused stem cell transplantation as consolidation treatment. Lenalidomide (20 mg, po, days 1–21, q28d) and prednisolone (10 mg, po, days 1–21, q28d) were used as a maintenance treatment to reduce the risk of lymphoma relapse. The reexamination every 3 months confirmed that the response was complete remission during the first year.

**FIGURE 2:**
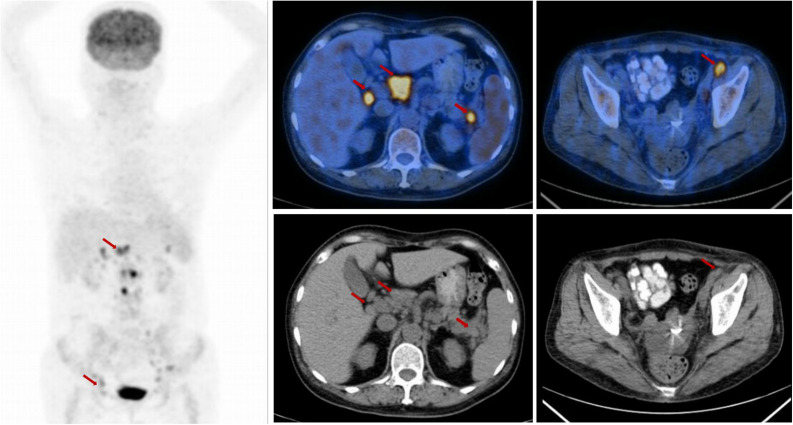
The positron emission tomography-computed tomography (PET-CT) scan at the time of relapse. The PET-CT scan showed elevated FDG uptake in the retroperitoneal and bilateral pelvic regions, (arrow).

**FIGURE 3:**
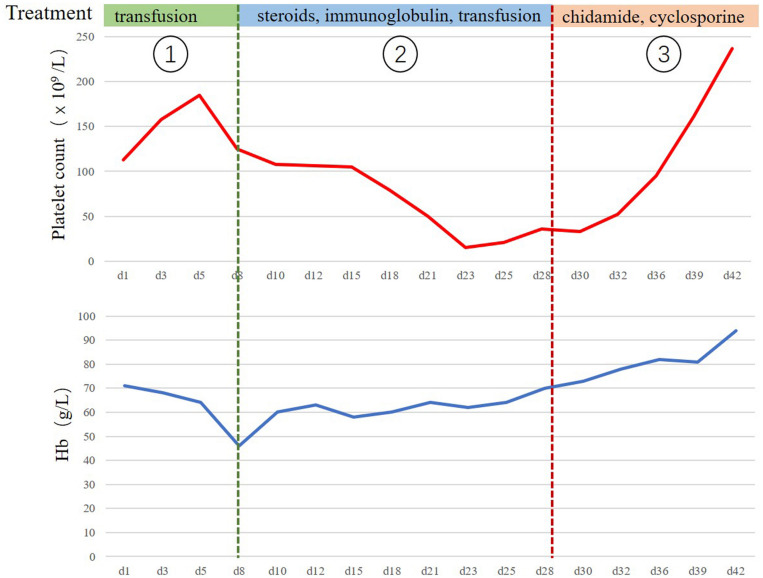
The clinical course of the treatment for relapsed/refractory angioimmunoblastic T cell lymphoma (AITL) with Evans syndrome. The clinical course from the occurrence of Evans syndrome to recovery. The continuous laboratory findings showed that anemia worsened quickly at the first stage, and the lowest number of Hb was 46 g/L. The main treatment was the transfusion of the washed red blood cells. The thrombocytopenia occurred 15 days later, and the lowest number of platelets was 15 × 10^9^/L. Refractory/relapsed AITL combined with secondary Evans syndrome was diagnosed at the second stage. The patient received the first-line treatments for Evans syndrome including prednisone and intravenous immunoglobulin. However, anemia and thrombocytopenia did not improve significantly. The combination of chidamide and cyclosporine were administrated at the third stage, and the anemia and thrombocytopenia gradually improved.

Unfortunately, she returned with fever, weakness after 12 months. The complete blood cell count showed anemia with hemoglobin (Hb) 71 g/L. The continuous laboratory findings showed that anemia worsened quickly, and thrombocytopenia occurred 15 days later. The lowest number of Hb was 46 g/L, and the lowest number of platelets was 15 × 10^9^/L. The direct Coombs test was strongly positive. The level of serum platelet-associated immunoglobulin G was significantly higher than normal. Elevated indirect bilirubin and lactate dehydrogenase were observed. The bone marrow biopsy was negative for lymphoma. The further ^18^FDG PET-CT scan confirmed that multiple FDG-avid enlarged lymph nodes in the retroperitoneal and bilateral pelvic regions (Figure 2). The patient could not receive re-biopsy of the enlarged lymph nodes in the retroperitoneal and bilateral pelvic regions considering the risk of bleeding because of thrombocytopenia. In view of the history of AITL, refractory/relapsed AITL combined with secondary Evans syndrome was diagnosed in this patient according to the diagnostic criteria reported by Jaime-Perez (8). However, the patient showed poor response to the first-line treatment for Evans syndrome including prednisone (2 mg/kg/day, po) for 2 weeks and intravenous immunoglobulin (0.4 g/kg/day, intravenous) for 5 days. Anemia and thrombocytopenia worsen progressively. Given the patient’s poor performance status, anemia, and thrombocytopenia, the second-line intensive salvage chemotherapy for refractory/relapsed AITL was not recommended. The combination regimen of chidamide (30 mg, po, biw) and cyclosporine were administrated. We set 2 mg/kg/d cyclosporine as an initial dosage and tapering by 50 mg every 3 weeks when the number of platelets and the red blood cells recovered to grade 2 anemia and thrombocytopenia. The direct Coombs test turned to be weakly positive 1 week later. The anemia and thrombocytopenia gradually improved after oral chidamide and cyclosporine, which was shown in Figure 3. Three months later, the laboratory findings including complete blood cell count, indirect bilirubin, lactate dehydrogenase, and serum platelet-associated immunoglobulin G recovered to normal, and the direct Coombs re-test was negative. The CT scan showed that the enlarged lymph nodes in the retroperitoneal and bilateral pelvic regions were shrinking significantly, and the response was complete remission. Five months later, chidamide (30 mg, po, biw) alone was used as the maintenance treatment up to now without serious adverse effects. The follow-up tests every 3 months were performed according to the NCCN practice guideline of T-cell lymphoma.

As of the write-up of this manuscript, the patient still lives a good quality of life without tumor progression till now and has already survived for more than 70 months.

## Discussion

The prognosis of AITL is unfavorable with the median overall survival less than 3 years and an estimated 5-year overall survival of only 30% (9). Many AITL patients are resistant to first-line chemotherapy or suffer from disease progression after a short-term remission with poor performance status, which results in the low tolerance to the second-line intensive salvage chemotherapy because of the increasing the risk of treatment-related adverse effects. It is a lack of established standard treatment for refractory/relapsed AITL till now. Thus, all refractory/relapsed AITL patients are potential candidates for new anti-tumor agents or combination regimens clinical trials.

Lenalidomide is an immunomodulatory drug, which has been confirmed to be effective and tolerable in relapsed/refractory AITL patients with the overall response rate of 30–40% and the complete response rate of 20–25% (10, 11). Lenalidomide showed efficacy and well tolerance in an 87-year-old patient with refractory ALTL to both gemcitabine and CHOP regimens in a case report (12). The above clinical trials and the case report revealed that lenalidomide alone or combination with other agents might be a considerable option for patients with relapsed/refractory AITL. In the present case, the addition of lenalidomide to CHOEP regimen improved the efficacy of this refractory AITL patient and obtained about 12 months progression-free survival with the maintenance treatment of lenalidomide and prednisolone.

Angioimmunoblastic T cell lymphoma patients usually presented with the manifestation of significant immune dysregulation including autoimmune hemolytic anemia, polyclonal hypergammaglobulinemia, and Evans syndrome (3). The precise mechanism remains unclear. AITL cells originate from follicular helper T cells, which can promote B-cell differentiation and proliferation through the interactions between T-cell receptor and B-cell receptor (13). The follicular helper T cells phenotype of AITL might be associated with the immune dysregulation through B-cell activation. In a retrospective study of 77 AITL patients, there were 19% of patients had autoimmune hemolytic anemia, 7% (5/76) patients with immune thrombocytopenic purpura, 2 patients with Evans syndrome, and 9% (6/66) patients with cold agglutinin hemolysis (3). Besides, all patients suffering from relapsed autoimmune cytopenia usually had AITL relapse simultaneous in the course of the disease. The AITL patients with the difficulty in treating autoimmune cytopenia were usually showed poor response to the treatment regimens of refractory AITL and had unfavorable prognosis (14). It is suggested that the treatment targeting AITL is the cornerstone of the treatment of immune dysregulation.

There is no standard treatment for Evans syndrome. Steroids are the first-line therapy considering that it can inhibit the ability of macrophages for clearing platelets and erythrocytes (15, 16). Intravenous immunoglobulins are recommended as a first-line treatment together with steroids in severely thrombocytopenic patients. They are also an important treatment in certain life-threatening circumstances for other autoimmune and hematologic diseases including immune thrombocytopenia, autoimmune hemolytic anemia, acquired hemophilia (17). In our case, both steroids and intravenous immunoglobulin were administrated when the patient was diagnosed with secondary Evans syndrome. Unfortunately, the patient showed a poor response to the first-line treatment with prednisone and intravenous immunoglobulin. Anemia and thrombocytopenia worsen progressively. The main reason for refractory Evans syndrome of this patient may be related to the uncontrolled refractory/relapsed AITL. The second-line treatment strategies include rituximab, mycophenolate mofetil, cyclosporine, and splenectomy (8). The immunosuppressive drug cyclosporine has been attempted in a small sample of patients with Evans syndrome since 1994 considering the mechanism of inhibiting the activation of T cells (18). Cyclosporine showed an excellent response to patients with Evans syndrome refractory to first-line therapy in many small series studies (19–21). Besides, there were some studies reported that cyclosporine following treatment with steroids or combined chemotherapy was effective in patients with relapsed AITL (22–25). However, it is very important to pay close attention to the adverse effects of the long-term use of cyclosporine including treatment-related renal toxicity and serious infection.

Chidamide is a novel orally histone deacetylase inhibitor (HDACi), which has inhibitory activity against HDAC1, 2, 3, 10 (26). It has shown activity against several types of tumors *in vitro* and *in vivo* (26–30). Chidamide might suppress T lymphoma cell growth and promote the apoptosis of T lymphoma cells though interfering with the binding between histone and DNA (31). Shi’ et al. study demonstrated the preferential efficacy and well tolerance of chidamide in 79 patients with refractory or relapse mature T-cell lymphoma (32). In this study, AITL and anaplastic lymphoma kinase-negative anaplastic large cell lymphoma revealed better response rates including overall survival rate and complete response, and AITL patients showed the best tolerance only with very few patients of grade 3/4 toxicity. Base on this study, chidamide was approved for the treatment of relapsed or refractory PTCLs by China Food and Drug Administration in December 2014. These results indicated that chidamide has significant efficacy and well tolerance in relapsed/refractory AITL patients with the overall response rate of 50%. In our report, the patient could not tolerate the second-line intensive salvage combined chemotherapy when she suffered from relapsed/refractory AITL combined with Evans syndrome because of the poor performance status, anemia, and thrombocytopenia. Therefore, the combination regimen of chidamide and cyclosporine were administrated considering the treatment targeting simultaneously both refractory/relapsed AITL and Evans syndrome with close attention to the blood cell counts and renal function. When this patient achieved complete remission, chidamide alone was used as a maintenance treatment to reduce the adverse effects of the long-term application of cyclosporine. Fortunately, this combination regimen was effective and well tolerant in this patient.

## Conclusion

In conclusion, refractory/relapsed AITL combined with Evans syndrome is a rare condition with a poor prognosis. There is no standard treatment strategy for refractory/relapsed AITL combined with Evans syndrome. Individual treatments should be based on the previous treatment and the response, the performance status, combined with the physician’s experience and close attention to the illness state of the patient during the period of the treatment. The combination of chidamide and cyclosporine may be an effective and tolerable regimen for the intractable AITL with Evans syndrome case and more observations are necessary to identify the efficacy and safety in the future.

## Data Availability Statement

The raw data supporting the conclusions of this article will be made available by the authors, without undue reservation.

## Ethics Statement

Written informed consent was obtained from the individual(s) for the publication of any potentially identifiable images or data included in this article.

## Author Contributions

GW and LZ designed the manuscript. TL and HP collected the data. YX, QL, and XL performed the analysis. FZ wrote the manuscript. LZ reviewed the manuscript. All authors contributed to the article and approved the submitted version.

## Conflict of Interest

The authors declare that the research was conducted in the absence of any commercial or financial relationships that could be construed as a potential conflict of interest.
